# A Single-Center 11-Year Experience of Surgical Management of Breast Cancer in Patients Aged 90 Years or Older

**DOI:** 10.7759/cureus.93656

**Published:** 2025-10-01

**Authors:** Sarah Joshi Puthur, Hudhaifah Shaker, Emily Coffey, Nabila Nasir, Mohammed Absar

**Affiliations:** 1 Department of Breast Surgery, North Manchester General Hospital, Manchester, GBR

**Keywords:** breast cancer management, disease free survival (dfs), general surgery and breast cancer, geriatrics population, overall survival (os)

## Abstract

Background

Surgery as a curative option for breast cancer is only offered to a fraction of women aged ≥90 years due to the lack of evidence supporting use of surgery in this patient group. This retrospective cohort study was undertaken to review patient outcomes following surgical treatment for breast cancer among women aged ≥ 90 years within our unit.

Methods

Retrospective case notes review and collection of clinicopathological data on 34 patients aged ≥ 90 years with early breast cancer who had surgery between 7th June 2007 and 31st July 2018 were carried out. Overall survival and disease-free survival were estimated using Kaplan-Meier survival analysis, with significance set to p <0.05.

Results

The median age of patients in the study was 90 years (range=90-101 years). The patients in this study were clinically fit and had Eastern Cooperative Oncology Group (ECOG) performance scores of 1 (67.6%) and 2 (32.4%). The mean tumour size was 33 mm (range=5-110mm).

A total of 28 patients (82.3%) underwent a mastectomy, two (5.8%) of which were bilateral. Six patients (17.6%) had a wide local excision, 13 axillary lymph node clearances were performed at the primary surgery, and none of the patients in the study developed any major post-operative complications.

A total of 25 patients (73.5%) had adjuvant endocrine therapy, and ten (29.4%) required adjuvant radiotherapy. None of the patients received adjuvant chemotherapy.

Over a median follow-up of 6.2 years, eight patients (26.4%) developed recurrence of disease, and 24 (70.5%) patients died at the time of data collection. Mean disease-free survival was 96.7 months (95% CI, 80.3- 113.2 months), and mean overall survival was estimated to be 68.5 months (95% CI, 35-84 months).

Conclusions

Breast cancer surgery is safe with minimal complications in medically fit women aged ≥ 90 years with early non-metastatic breast cancer and offers good local control.

## Introduction

Breast cancer is one of the most common cancers affecting women in the UK, with around 55,500 new cases diagnosed annually [1]. Age is the strongest risk factor for breast cancer, and hence, the highest breast cancer incidence rates are seen in older women [1]. According to Cancer Research UK (2021), 3.2% of new cases of breast cancer were diagnosed in women aged ≥90 years between 2016-2018. As a result of advances in healthcare, especially public health, the number of people aged ≥65 years in the UK has increased from 9.2 million in 2011 to over 11 million in 2021 [2]. It was estimated that there were around 350,000 women aged ≥90 years in the UK in 2023 [3]. Thus, the elderly represent a rapidly expanding segment of the UK population. Advances in modern medicine have led to significant improvement in the prognosis for women diagnosed with early invasive breast cancer since the 1990s, and most breast cancer patients can expect to be long-term cancer survivors [4].

Current guidelines on the management of breast cancer

Surgery is the mainstay of treatment of both early and locally advanced breast cancer and may involve surgery to the breast (wide local excision, mastectomy) and axilla (sentinel lymph node biopsy, axillary lymph node clearance) with or without breast reconstruction [5]. The National Institute for Health and Care Excellence (NICE) Breast Cancer Quality Standard states that women with early invasive breast cancer should be offered surgery irrespective of age [5]. A joint paper published by the European Society of Breast Cancer Specialists (EUSOMA) and the International Society of Geriatric Oncology (SIOG) recommended that surgery should be the primary choice of treatment in fit elderly women with breast cancer who have an expected life expectancy of greater than five years [6].

Surgery and elderly patients

Although UK national guidelines recommend surgery as a treatment option for women of all ages, only a fraction of patients aged ≥90 years are offered surgery as a curative treatment option. According to the most recent UK National Audit of Breast Cancer in Older Patients (NABCOP) in 2022, only 55% of women aged ≥80 years have surgery for early invasive breast cancer compared to 97% for women aged 50-59 years [7]. The NABCOP report does not clarify the percentage of women aged ≥90 years who undergo surgery for early invasive breast cancer [7]. One of the primary reasons for the low rates of surgery in patients aged ≥90 years is that most clinicians utilise patients’ chronological age to decide on treatment strategies and associate advanced age with higher operative risks [8]. Other reasons for low rates of breast cancer surgery include patient co-morbidities that preclude anaesthesia and patient preference [8].

Lack of evidence supporting the use of surgery

Multicentre studies such as the Bridging the Age Gap in Breast Cancer study have demonstrated benefits to offering women aged ≥70 years surgery for breast cancer [9]. However, there is a lack of evidence in medical literature exploring patient outcomes following surgical treatment for breast cancer among the cohort of women aged ≥90 years, which further compounds the low rates of breast cancer surgery. At present, there is limited data on tumour characteristics, treatment choices, overall survival (OS), and disease-free survival (DFS) in breast cancer patients aged ≥90 years [10, 11]. This is largely due to low enrolment of patients aged ≥90 years in clinical trials [11, 12]. Hence, due to a lack of evidence supporting the use of surgery as a curative option in this patient demographic, surgery is not routinely offered.

Given the paucity of evidence in current medical literature on patient outcomes following surgery for breast cancer in women aged ≥90 years, this study was undertaken to evaluate the safety and efficacy of surgical treatment in women aged ≥90 years by assessing postoperative complications, DFS, and OS.

## Materials and methods

Study design and sample

This retrospective cohort study was carried out within the Breast Surgery Unit at North Manchester General Hospital, Manchester University NHS Foundation Trust. Data collection was conducted between March 2018 and July 2018 using patients’ hospital records and a pathology database which is searchable by age. All women aged ≥90 years diagnosed with early breast cancer and treated with surgery between 7th June 2007 and 31st July 2018 at the Breast Surgery Unit were identified and included in this study. Patients with metastatic disease were excluded from the study. A case note review was performed using electronic patient and pathological records. Prior to surgery, all patients underwent standard physical examinations, preoperative assessments, and staging according to the protocol used at the Breast Surgery Unit. Only patients with Eastern Cooperative Oncology Group (ECOG) scores of ≤2 were considered medically fit and offered surgery at the unit. Patients with ECOG scores >2 were excluded from this study.

Clinicopathological data

Data was collected on patients’ tumor characteristics that included tumor type (WHO classification), tumor grade, oestrogen receptor (ER) status, human-epidermal growth factor receptor 2 (HER2) status, and Ki67 expression, as well as lymph node status. Patients were also assessed on their co-morbidities and frailty using the Charlson Co-morbidity index (CCI), number of co-morbidities, and ECOG performance scale.

Oncology treatment

Electronic patient records were reviewed to obtain data on the type of breast cancer surgery patients underwent, the type of axillary surgery, and adjuvant therapy. Breast cancer surgery was classified into either wide local excision or mastectomy (unilateral or bilateral) with or without breast reconstruction. Axillary surgery in this study was defined as either a sentinel lymph node biopsy (SLNB) or an axillary node clearance. Adjuvant therapies included in this study were further grouped into adjuvant radiotherapy, adjuvant endocrine therapy, or adjuvant chemotherapy. The present study also reviewed postoperative complications that patients developed during the study period, such as seroma, wound infections, hematoma, and any unplanned return to theatre. Wound infections were defined as surgical wound infections that required a course of antibiotics, and return to theatre was considered in patients who had any unplanned postoperative surgery in this present study.

Survival and statistical analysis

The primary outcomes of this study were to determine the OS, DFS and recurrence rate among medically fit patients aged ≥90 years who underwent breast cancer surgery. Recurrence was defined as any local (breast), regional (axillae), or distant recurrence after breast cancer surgery. Patients who died during the study were identified using the Manchester Register Office, which was linked to patients’ electronic hospital records. The cause of death was determined using the patient’s hospital records. All patients were followed up for a minimum of six months after their initial surgery. OS and DFS were estimated using Kaplan-Meier survival analysis. Statistical analysis was performed using JASP (version 0.95.0) software with statistical significance set to p <0.05.

## Results

Clinicopathological details

A total of 34 female patients aged ≥90 years underwent surgery for early breast cancer during the 11-year period. The Breast Surgery unit treated 3,276 patients between 2007-2018, and hence, these patients represented 1% of all breast cancers treated during this time period within the unit. The median age of patients at diagnosis was 90 years (range 90-101 years). Hypertension (32.3%) and coronary artery disease (32.3%) were the most common comorbidities in the patient cohort (Table 1). The patients in this study were clinically fit and had ECOG scores of 1 (67.6%) and 2 (32.4%). The Non-age-adjusted Charlson Comorbidity Index for the majority of the patients was between 0-1 (79.4%). Age was excluded as a factor for calculating the CCI in this cohort, as all patients in this study were aged ≥90 years (Table 1).

**Table 1:** Co-morbidities in patients

Co-morbidity	n (%)
Atrial fibrillation	6 (17.6)
Asthma	1 (2.9)
Coronary artery disease	11 (32.3)
Chronic kidney disease	4 (11.8)
Congestive heart failure	2 (5.9)
Chronic obstructive pulmonary disease	1 (2.9)
Cerebral vascular accident	3 (8.8)
Dementia	4 (11.8)
Diabetes	1(2.9)
Diverticular disease	1(2.9)
Gastroesophageal reflux disease	1(2.9)
Hypertension	11 (32.3)
Thyroid issues (hyperthyroidism or hypothyroidism)	4 (11.8)
Osteoarthritis	7 (20.6)
Other cancers	1(2.9)
Parkinson’s disease	1(2.9)
No. of co-morbidities seen in patients:	
No co-morbidities	8 (23.5)
1	7 (20.6)
2	8 (23.5)
≥3 co-morbidities	11 (32.3)
Non-age-adjusted Charlson Comorbidity Index	
0	20 (58.8)
1	7 (20.5)
2	2 (5.9)
3	3 (8.8)
≥4	2 (5.9)

Mean tumour size was 33mm (range 5-110mm). A total of 13 cancers (38%) were ER-negative, two (6%) were HER2-positive, and 12 (35%) were grade 3 (Table 2). Eight (24%) patients had positive axillary lymph node metastasis. The most common type of breast cancer among this cohort of patients was invasive ductal carcinoma, representing 64% of diagnoses.

**Table 2:** Clinicopathological and oncological treatment details of the study participants

Parameter	n (%)
Invasive grade	
1	6 (18)
2	16 (47)
3	12 (35)
ER status	
Positive	21 (62)
Negative	13 (38)
Ki67 expression	
< 20% expression	25 (74)
>20% expression	9 (26)
Lymph node status	
Positive	8 (24)
Negative	26 (76)
HER 2 status	
Positive	2 (6)
Negative	32 (94)
Type of surgery	
Unilateral mastectomy	26 (76.5)
Bilateral mastectomy	2 (5.8)
Wide local excision	6 (17.6)
Breast cancer sub-type	
Invasive ductal carcinoma	22 (64)
Invasive ductal carcinoma + invasive lobular carcinoma	3 (9)
Mucinous	3 (9)
Invasive lobular carcinoma	1 (3)
Tubular	1 (3)
Ductal carcinoma in situ	1 (3)
Isolated tumour cells	1 (3)
Medullary	1 (3)
Squamous	1 (3)
Type of axillary surgery performed at primary surgery	
Axillary node clearance	13 (38.2)
Sentinel lymph node biopsy	11 (32.3)
Neither performed at primary surgery	11 (32.3)
Type of adjuvant therapy	
Adjuvant endocrine therapy	25 (73.5)
Adjuvant radiotherapy	10 (29.4)
No adjuvant therapy	13 (38.2)

Oncological treatment

A total of 28 (82.3%) patients underwent a mastectomy, two (5.8%) of which were bilateral. Six (17.6%) patients had a wide local excision (Table 2). A total of 13 axillary lymph node clearances were performed at the primary surgery (Table 2). None of the patients in this study had any reconstructive surgery.

The overall rate of patients with at least one postoperative complication was 20.6%. Three (9.6%) patients developed seroma, two patients (6.5%) developed wound infections, one patient (3.2%) developed a hematoma, and one patient (3.2%) developed temporary numbness of the left upper arm. All the patients who developed postoperative complications were managed conservatively, and none of the patients required any unplanned return to theatre. The 30-day postoperative mortality rate among this cohort was zero.

A total of 25 (73.5%) patients had adjuvant endocrine therapy; 10 patients (29.4%) required adjuvant radiotherapy, and 13 patients (38.2%) did not receive any adjuvant therapy (Table 1). None of the patients received adjuvant chemotherapy.

Recurrence and survival

Median follow-up was 78 months (interquartile range=46.5-95 months). The recurrence rate in this cohort of patients was 26.4% (n=8). In total, 12 recurrence events occurred in these eight patients, with some patients developing disease at multiple sites. Nine percent of patients (n=3) developed local recurrence while 20.6% of patients (n=7) developed distant metastases. There were two cases (6%) of axillary recurrence, and one of these patients was managed surgically with axillary lymph node clearance. The remaining patients who developed recurrence were managed with supportive care only. Among the patients with recurrence of disease, five patients (14.7%) had received adjuvant radiotherapy.

**Figure 1:**
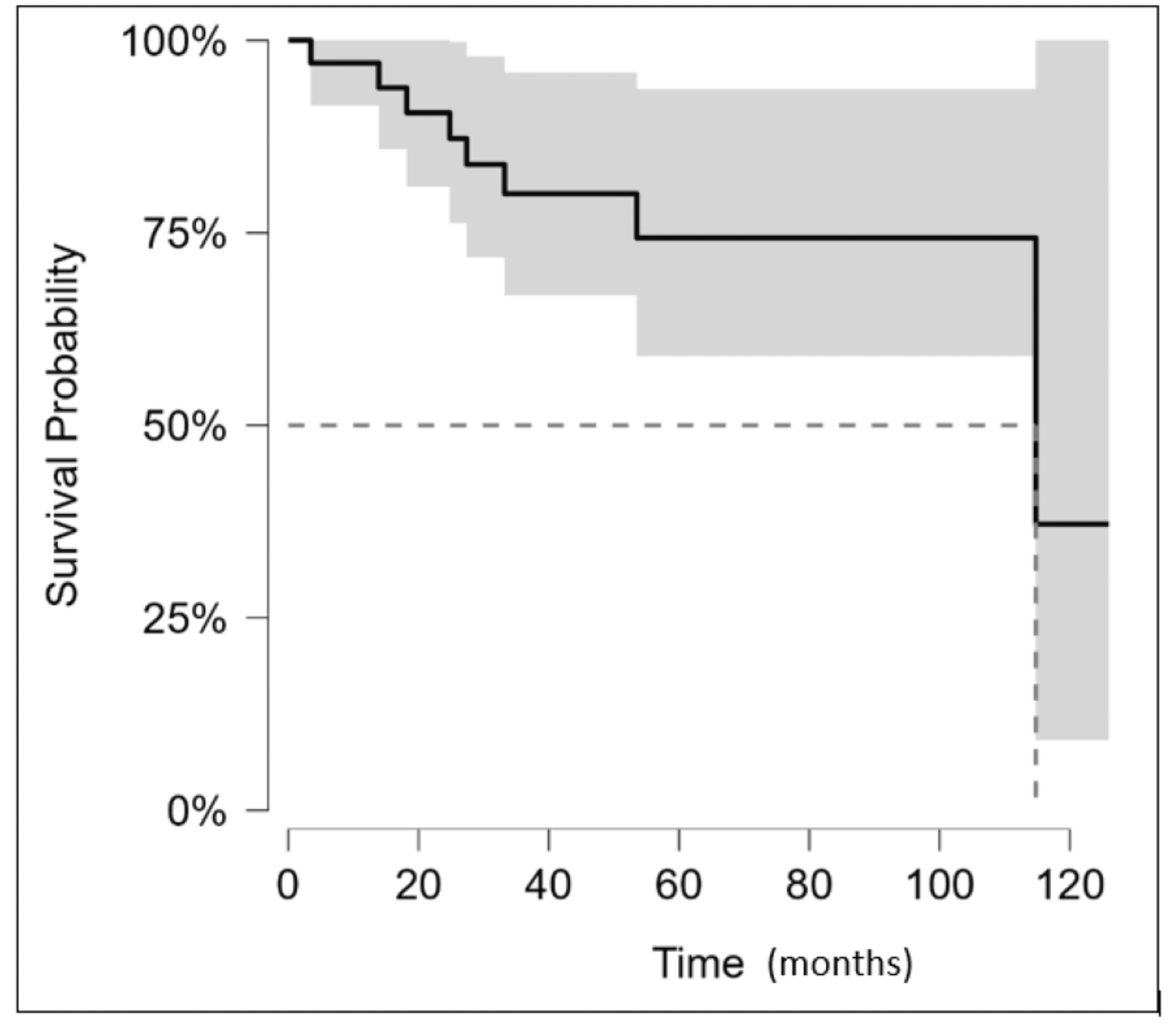
Kaplan-Meier curve for DFS in the patients included in the study. Note: DFS was calculated from the time of the primary surgery to the time of first recurrence. If no recurrence of disease was documented, patients were censored at the date of the last known follow-up. Median DFS was 114.8 months (indicated using the dotted lines), and the confidence interval is denoted by the shaded area. DFS: disease-free survival

Over a median follow-up of 78 months, 70.5% of patients (n = 24) had died at the time of data collection, of whom four patients (11.8%) died within 12 months of their breast cancer surgery. Cause of death was known in 47% of patients, and six of these patients (37.5%) were attributable to their breast cancer. Other causes of death in patients were infection (37.5%), other malignancies (18.8%), and one patient (2.2%) died due to an ischaemic event. Based on the Kaplan Meier survival analysis, mean DFS was 96.7 months (95% CI, 80.3-113.2 months) and median DFS was 114.8 months (Figure 1, Table 3). Similarly, the mean and median OS were estimated to be 68.5 months (95% CI, 35-84 months) and 67 months, respectively (Figure 3, Table 4).

**Table 3:** Number of patients at risk at specified time points (corresponding to Figure 1)

Months	0	20	40	60	80	100	120	140
At risk (censored)	34 (0)	28 (4)	16 (8)	13 (2)	4 (10)	2 (1)	1(0)	1(1)

**Figure 2:**
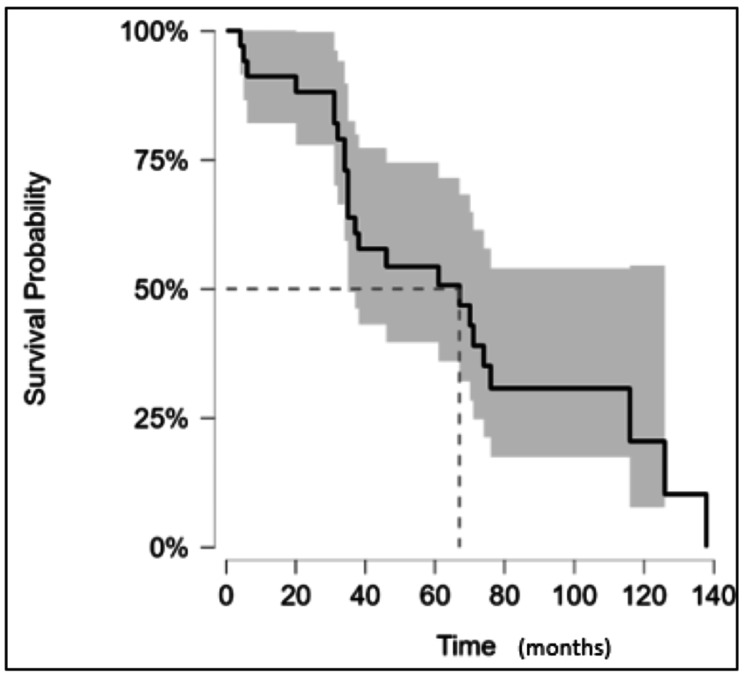
Kaplan-Meier curve for OS in the patients included in the study. Note: OS was calculated from the time of the primary surgery to the time of death. If not deceased, patients were censored at the date of the last known follow-up. Median OS was 67 months (indicated using the dotted lines) and confidence interval is denoted by the shaded area. OS: overall survival

**Table 4:** Number of patients at risk at specified time points (corresponding to Figure 2)

Months	0	20	40	60	80	100	120	140
At risk (censored)	34 (0)	30 (1)	19 (0)	15 (3)	6 (4)	3 (2)	2 (0)	0(0)

## Discussion

This study demonstrates that focused surgical treatment provides optimal DFS in women aged ≥90 years with early breast cancer who are medically fit for surgery.

One of the main reasons for the outcomes seen among the patients in this current study may be due to the presence of less aggressive variants of breast cancer among elderly women. In older women, breast cancer phenotypes are more favourable and with lower proliferative rates, ER- and PR-positive status, and lower rates of HER2 overexpression [12, 13]. Prevalence of breast cancer subtypes that confer a poor prognosis, such as triple-negative breast cancer, is only seen in 15% of elderly patients [13]. This was reflected in our findings as only two patients had the HER2-positive subtype, and none of the patients had triple-negative cancer.

Breast cancer surgery is also low-risk compared to surgeries for other types of cancer. In a global, multicentre prospective cohort study undertaken by GlobalSurg Collaborative, breast cancer surgery had the lowest mortality rate of 0.2% compared to 5.4% and 3.3% seen in gastric and colorectal cancer surgeries, respectively [14]. Among elderly patients aged ≥70 years, the overall mortality rate after breast cancer surgery is <1% and the systemic complication rates are roughly 2% [15, 16, 10]. Anaesthesia-related complication rates are low in breast cancer surgery, and local anaesthetics can be used as an effective alternative for general anaesthesia in frail elderly patients [17]. In the present study, none of the 34 patients who underwent breast cancer surgery developed significant postoperative complications or died from complications related to the surgery.

Studies have shown that surgery is a crucial factor for improving OS and cancer-specific survival in breast cancer patients, irrespective of age, tumour, stage, ER/PR, and HER2 status [8, 18]. A Cochrane systematic review concluded that surgery with endocrine treatment was superior to primary endocrine treatment alone for local control of breast cancer in ER-unselected patients aged ≥70 years [19]. However, there was no statistically significant benefit to offering surgery for increasing OS [19]. Similar results were obtained in a randomised trial of one hundred fifty-three fit women aged ≥70 years carried out by Johnston et al. (2011), where failure rates of local control in the group treated with primary endocrine therapy (tamoxifen) alone were 43% compared to 1.9% in the group treated with mastectomy and adjuvant endocrine therapy [20]. Several previous randomised control trials, including the GRETA trial, have reported high rates of failure of local control in groups receiving primary endocrine therapy alone [21-23]. Additionally, in our study, where the mean age of patients was 92 years, medically fit patients benefited from optimal DFS (mean DFS of 96.7 months) and OS (mean OS of 68.5 months) when offered surgery. In England, the average life expectancy of a 90-year-old woman with minimal co-morbidities is 4.5 years or 54 months [24]. Hence, the patients in our study had OS rates that were comparable to the life expectancy observed in women aged ≥90 years in the general population.

It is important to note that very few studies have reviewed OS and DFS following breast cancer surgery in patients aged ≥90 years. Contrary to the current NICE guidelines, only a fraction of patients aged ≥90 years are offered breast cancer surgery, largely due to a lack of evidence favouring surgery in this patient demographic. Participants aged ≥90 years are often excluded from clinical trials exploring the benefits of surgery, as increasing age is often associated with increased frailty. While most elderly patients have multiple co-morbidities that preclude them from having surgery, it is crucial to appreciate the heterogeneous nature of the elderly population, which includes patients who are fit for surgery with a long life expectancy as well as patients with increased frailty and short life expectancy [25]. Thus, chronological age alone is a poor predictor of morbidity/mortality after breast cancer surgery and excludes patients aged ≥90 years who are medically fit and can tolerate surgery. Instead, clinicians should consider patients’ pre-existing co-morbidities, functional status, and cognitive impairment to decide treatment strategies [26]. This can be achieved by using tools such as the Comprehensive Geriatric Assessment (CGA) to assess frailty, predict morbidity/mortality, and determine eligibility for breast cancer surgery in patients aged ≥90 years who may be good candidates for breast cancer surgery [26].

Given the limited life expectancy among breast cancer patients aged ≥90 years, the ultimate goal of breast cancer management in elderly women should be focused towards achieving good local control, minimising functional loss, and improving quality of life. In our study, only 17.6% of patients developed metastasis, and the majority of patients had good local control. Achieving good local control in a patient demographic with low-risk breast cancer subtypes, favourable tumour biology, and low proliferation rates can reduce the need for further unnecessary surgical interventions such as SLNB and associated complications. At present, the ‘Choosing Wisely’ initiative by the Society of Surgical Oncology (SSO) has recommended the omission of routine SLNB in low-risk elderly breast cancer patients [27]. Arguments in favour of omission of routine SLNB in elderly women include no benefit in terms of locoregional control, does not provide additional information to guide systemic therapy, and a positive SLN status was not significantly associated with OS [27, 28]. Additionally, SLNB confers risks of complications such as lymphedema, arm swelling, pain, and reduced shoulder mobility, which can result in functional impairment in these patients [27]. In our study, 11 patients (32.3%) had neither SLNB nor axillary node clearance; six patients (17.6%) declined any form of axillary surgery, while five patients (14.7%) had clinically node-negative, hormone receptor-positive and HER2 receptor-negative breast cancers. Since these five patients had favourable tumour profiles, axillary surgery was omitted in these patients in accordance with the ‘Choosing Wisely’ initiative.

The wider implications of this study are to highlight the undertreatment of breast cancer in medically fit patients aged ≥90 years and support the use of surgery as an option for achieving good local control in these patients. Presently, there is a paucity of evidence in current medical literature that explores survival outcomes following breast cancer surgery in patients aged ≥90 years, and our study is one of the few studies that reviewed survival outcomes in this patient demographic. Hence, we hope our findings would not only pave the way for future research involving this patient demographic but also encourage clinicians to weigh treatment decisions based on patient co-morbidities rather than chronological age alone.

The authors recognise the limitations in this study. Firstly, the retrospective nature of the study may introduce biases in data collection. Secondly, the study reviewed postoperative and survival outcomes following breast cancer surgery using a small sample of medically fit women aged ≥90 years with early breast cancer at a single institution. Moreover, the study did not include a comparison of survival outcomes between women who were treated with surgery versus primary endocrine therapy alone. We recognise that a stronger argument in favour of supporting breast cancer surgery to provide optimal local control in medically fit elderly women can be made if further research is undertaken to compare the recurrence rates and survival outcomes against patients aged ≥90 years treated with endocrine therapy alone.

## Conclusions

Breast cancer patients aged ≥90 years are often under-treated because increasing age is often associated with increasing frailty. The under-treatment of these patients is further compounded by the lack of research that supports the use of surgery for local control of breast cancer in patients aged ≥90 years. The findings of this study demonstrate that breast cancer surgery is safe and offers good local control in a subset of medically fit patients aged ≥90 years. Clinicians need to recognize the heterogeneous nature of the elderly population and consider patient co-morbidities and fitness for surgery when deciding treatment strategies. The aging population is a rapidly growing segment of the population, and hence, it is of paramount importance that further research using a larger study sample is carried out to review local recurrence rates and survival outcomes in medically fit patients aged ≥90 years with early breast cancer and treated with surgery.
